# Annotations

**Published:** 1888-05-26

**Authors:** 


### ANNOTATIONS.

**Asylum Attendants.**

WHILE so much attention and care has been expended on raising the status of hospital nurses in proportion to the increasing skill and thought demanded of them, very little attention has been given to the equally important question of the attendants of the insane, on whom an even greater responsibility devolves. Firm nerves and a sympathetic soul are needed yet more, if that be possible, in dealing with the insane than with the sick; and while the majority of hospital patients are such as need attention only to their bodily wants, insane patients want an exceptional degree of consideration for their mental state, not only as it is, with the "sweet bells jangled out of tune," but as it was in health. To a lunatic who has intervals of sanity, or whose aberrations do not affect the original culture and refinement of his mind, nothing can be more distressing and more injurious than the society of an attendant who, however well-intentioned, is coarse and illiterate. It must be remembered that an asylum patient's servant is more to him than the doctor. He, indeed, sees the latter daily, and exchanges a few words with him, but his servant is *par excellence*, his friend, companion, and confidant. Moreover, the servant must be capable, on occasion, of proving the master, and it is therefore essential that his rule should be of a kind that will command obedience by force of respect rather than of fear. Everybody knows how children may suffer irreparable injury from being left to the care of a nurse who, though thoroughly well meaning, is mentally thick-skinned and dull, who frightens where she should soothe and yet loses the power that terror gives her by lack of real strength of character. Such persons are even more unfit for the purpose of tending the grown-up children whom we term insane. Those of us who know how much culture and artistic and intellectual power are often to be found within the walls of asylums cannot but pity those who, confined there simply for lack of mental ballast to keep those qualities in their proper place, are reduced to the companionship of attendants so ignorant as to be almost intolerable to any one of moderate education. In many private asylums we now find lady attendants, who, being educated and refined, help to create something worthy of the name of society, and make the asylum an endurable—often pleasant—home for female patients; but it is difficult to find any counterpart to this in the male wards. The salaries offered—£35, £40 to £50 a-year—are not large enough to offer any inducements to even the higher class of men servants, and let the resident doctors do what they will—and it cannot be denied that they do much for the welfare and amusement of those entrusted to their care—they are too few to fill up the want we speak of. In cases where large sums are paid for the maintenance of the patient, the employment of old soldiers, who from their supposed capacity for discipline, are favourite candidates for these posts, and uneducated men generally, as attendants, is wholly inexcusable. A fair proportion of the sum received should go towards providing the patient with a proper servant. The illiterate, dull, or tyrannical attendant assigned to him, must often aggravate a patient's sufferings and retard his recovery.

**Faults on both Sides.**

THE dispute between the honorary medical officers and the Committee of Management at the Dorset County Hospital has, it may be hoped, at last come to an end. The doctors have consented to bow to the decision of the Governors. We do not agree with the stale platitude, that there are *always* faults on both sides. It is not true in every case, and sometimes it is flagrantly false. On this occasion, however, it would seem that a little more "sweet reasonableness" on either side would have prevented a very great deal of painful envy and a very considerable public scandal. So far as appears, the matron has claimed as a right the liberty of going round the wards with the doctors whenever she might choose, and also the freedom of being present at all surgical operations at her own discretion. The honorary medical officers, on the other hand, seem to have wished to have more control over the wards and the operation theatre than was compatible with the matron's claim. The majority of the committee took the part of the matron. That seems to have instituted the real gist of the case, stripped of all embellishments. From a former report we were led to the conclusion that the matron insisted on receiving all nursing instructions from the medical men herself, and conveying them at second-hand to the nurses in charge. That was clearly a mistaken policy, and might in many cases have resulted in injury and danger to patients, and of such a line of action we thought it necessary to express emphatic disapproval. The rule should be, that wherever practicable the nurse who is to be placed in charge of any given case should receive her instructions directly from the physician or surgeon who has care of the patient. But the desire that the matron shall be present in the wards and hear what the doctor has to say about the nursing is very different from the demand that she only shall be present, and to her alone shall all the instructions about nursing be given, and that she shall retail them in her own fashion to the nurse in charge. Just as we formerly insisted that the doctor himself should give his instructions to the nurse who was responsible for the case, so do we now insist that as the matron is a trained nurse, and should properly hold herself charged with the duty of directing each subordinate nurse, she should have the opportunity of making herself familiar with the necessities and details of each case. Otherwise, how can she exercise that general and special control which constitute the most important part of her duties? It may be hoped that a little good sense, a little good feeling, and a little mutual regard will be sufficient to restore and maintain perfect peace for the future.

**The Public Demands Inquiry.**

A LARGE number of our contemporaries have entered upon the discussion raised by Mr. Burdett-Coutts at the Hospitals Association with regard to the payment by hospital patients of a portion of the cost of their treatment. Lord Randolph Churchill's vigorous philippics at St. Mary's have added intensity to the earnestness already manifested with regard to this and other hospital questions. What the public plainly sees are the great and prominent facts of the case. Small expediencies and local difficulties are held of no account. The great facts are, that the hospitals are in difficulties, that more than a fifth of their beds are always empty, not for lack of patients, but for lack of means. It is also seen that hundreds of patients are treated at hospitals who, although they may be more or less limited in means, yet are not absolutely poor. They could afford to pay, if not the whole cost of their treatment, at least something towards it. "Why," says the badgered and baited philanthropist, "why should I be worried every day in the week, and by almost every post, to help this hospital or that dispensary, or the other truss society, when I know that thousands of pounds are given away every year to thousands of persons who are no more straitened in circumstances than I am?" The badgered philanthropist has right on his side. There is no sufficient reason why he should be so pestered beyond endurance, at any rate until it is proved that no money is wasted at hospitals on those who are not poor. The hospital world as a whole makes no attempt to deal with the question. Its only cry is "Give, give." Begging is easier than a statesmanlike grappling with difficulties. The hospital world as a whole must make some effort to show not only that it can beg persistently, but also that it can spend wisely. It is absolutely astonishing that the only answer given to the almost universal criticism of the press is folded hands and a despairing look. Mr. Burdett-Coutts returns to the attack on June 20th. The defenders of the *status quo* must turn out in numbers and strength, or judgment will inevitably be given against them.

